# Limitations of the use of the MP-RAGE to identify neural changes in the brain: recent cigarette smoking alters gray matter indices in the striatum

**DOI:** 10.3389/fnhum.2014.01052

**Published:** 2015-01-28

**Authors:** Teresa R. Franklin, Reagan R. Wetherill, Kanchana Jagannathan, Nathan Hager, Charles P. O'Brien, Anna Rose Childress

**Affiliations:** Department of Psychiatry, Perelman School of Medicine at the University of PennsylvaniaPhiladelphia, PA, USA

**Keywords:** voxel based morphometry, ventral striatum, cigarette smoking, gray matter volume, longitudinal, T1-weighted, MP-RAGE

The magnetization-prepared rapid gradient-echo (MP-RAGE) T1-weighted high resolution structural MRI is a mainstay tool used to identify morphometric biomarkers of disease conditions, progression and treatment effects despite a critical limitation: the relaxation signal on which inferences are based is nearly indistinguishable for gray matter vs. blood flow (Lu et al., 2004; Wright et al., 2008). Thus, apparent reported morphometric findings might be at least partially related to transient changes in blood flow or other physiological signals.

Consistent with this technical limitation, using a standard analysis technique, voxel based morphometry (VBM), we recently reported that a single dose of a medication had “apparent” effects on T1-weighted MRIs (Franklin et al., 2013). Specifically, we observed medication-induced decreases in gray matter volume in the anterior cingulate and other regions that overlapped with changes in brain blood flow (perfusion). Similarly, others have shown effects of medication on T1-weighted scans that are likely transient. For example, acute levodopa administration altered gray matter indices on T1-weighted images in the midbrain (Salgado-Pineda et al., 2006). Further, in a well-controlled longitudinal VBM study of patients with attention deficit hyperactivity disorder (ADHD), Hoekzema et al. (2014) showed that stimulant drugs trigger transient volumetric changes in the ventral striatum, challenging previous VBM studies suggesting that these changes were an intrinsic feature of the disorder (Hoekzema et al., 2014). These examples highlight the importance of employing appropriate experimental conditions to ensure that morphometric findings are actually revealing long-term changes that are unrelated to transient states. Specifically, longitudinal investigations into the effects of a medication on neural structure using VBM may want to include a group that is administered an acute dose of the medication.

The findings of altered gray matter induced by acute pharmacological manipulations highlighted above led us to hypothesize that cigarette smoking, which delivers the psychostimulant, nicotine, to the brain may transiently alter the MP-RAGE, based on the T1-weighted images. Thus, we examined T1-weighted structural MRIs acquired in a within-subjects design in 39 otherwise healthy nicotine-dependent individuals. Subjects meeting our standard MRI-specific inclusion/exclusion criteria (Franklin et al., 2013, 2014) provided written informed consent to participate in the study, which adhered to the Declaration of Helsinki and was approved by the University of Pennsylvania Institutional Review Board. Screening procedures included a medical history, physical examination, and a psychological assessment. Subjects participated in two counterbalanced scanning sessions that occurred on separate days. For condition “Smoke” subjects smoked a cigarette to satiety approximately 45 min prior to obtaining structural MRI data. For condition “No smoke,” subjects smoked a cigarette and then remained abstinent for approximately 5 h prior to data acquisition. To obtain quantitative cerebral blood flow (CBF) maps, 5 min resting baseline pseudo-continuous arterial spin labeled perfusion fMRI scans were also acquired under both conditions. Nicotine's terminal half-life is approximately 2 h; thus, conservative estimates might place the onset of withdrawal symptomatology within the first 2 h after last smoking (Benowitz et al., 1982). Thus, 5 h of deprivation was chosen to ensure that the subjects would be experiencing withdrawal from nicotine. During the “No smoke” condition, carbon monoxide (CO) levels were acquired after the baseline cigarette and prior to scanning. Abstinence was confirmed by monitoring the subjects and was biochemically confirmed by observing reductions in CO from baseline to time of scan acquisition. Structural data were analyzed using statistical mapping software (SPM8) and its available VBM tool with diffeomorphic anatomical registration using exponentiated lie algebra (DARTEL). Statistical parametric maps were generated to perform between-condition comparisons (paired *t-test*) using the gray matter tissue segmentation output by DARTEL. To obtain measures of brain blood flow mean CBF maps were calculated for each condition and between-condition comparisons (paired *t*-test) were performed. Image acquisition, data processing and analysis methods have been previously published (Franklin et al., 2013, 2014).

Once again, we report that transient state impacts gray matter indices. Compared to not smoking for several hours, recent smoking altered the T-1 weighted MP-RAGE, bilaterally in the striatum (see Figure 1 and http://www.franklinbrainimaging.com/LimitationsofMPRAGE/, which provides all images from all 3 planes). Differences were not observed in any other brain regions. Additionally, as in our previous study, the effects overlapped with changes in blood flow (Figure S1 shows the results from the MP-RAGE and blood flow overlain on the same template. These results unambiguously demonstrate that the use of VBM to identify morphometric biomarkers of disease conditions, progression and treatment effects must employ appropriate experimental conditions to ensure that morphometric findings are not related to acute states that may be introduced by drug or medication, but are indeed revealing long-term change. Importantly, the use of medications or substances (e.g., alcohol, cigarettes, concomitant medications) that affect the brain should be controlled (either through matching or statistical co-variation) across groups that are being compared. Third, these results show that the MP-RAGE should be acquired at the initiation of a scanning session, given the potential for it to be altered by subsequent activities (tasks, pharmacological manipulations) that occur over the course of scanning. These findings also underscore the crucial and immediate need to develop neuroimaging tools that can uniquely capture changes in neuronal structure dissociable from those related to blood flow or other physiological signals.

**Figure 1:**
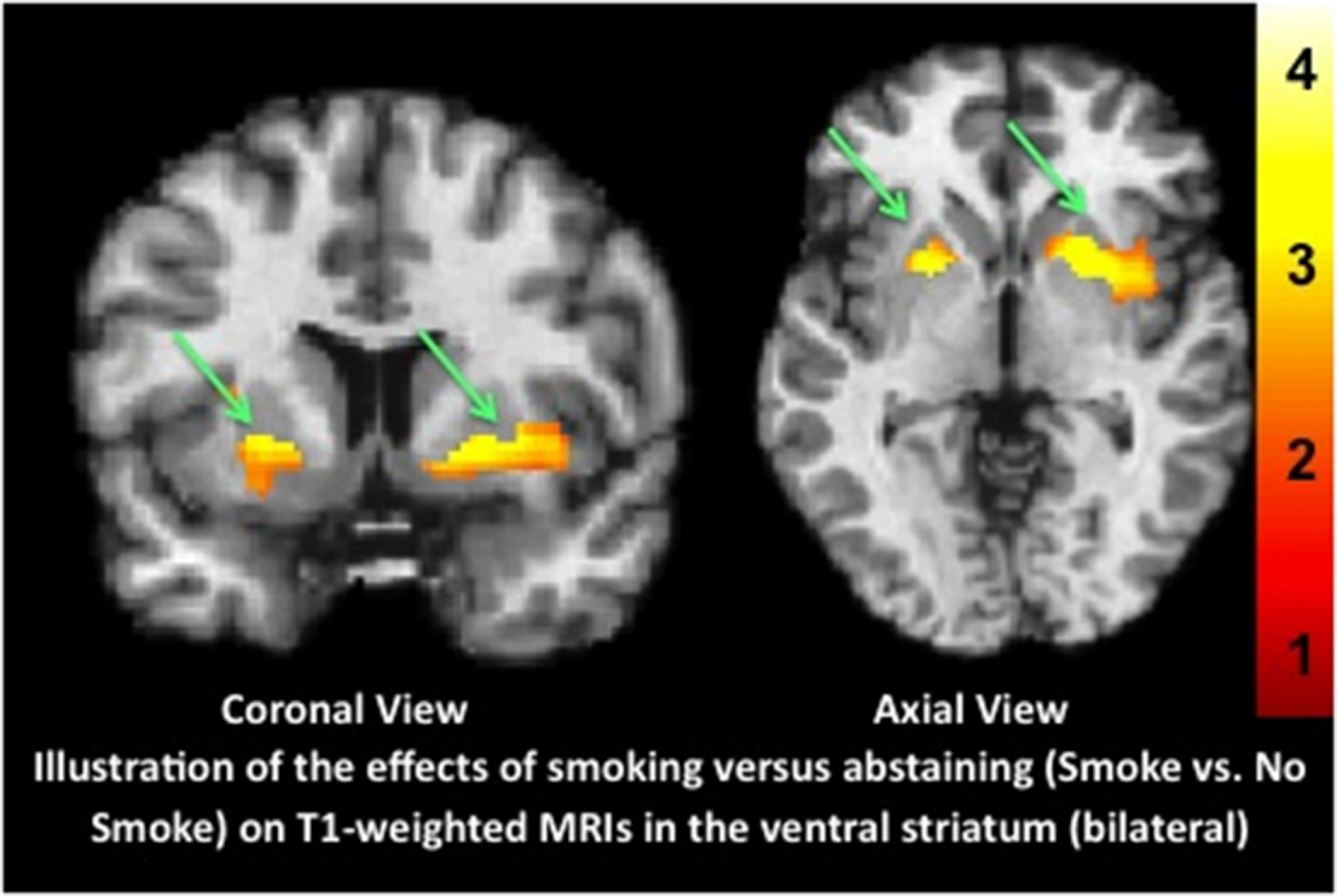
**Illustration of the effects of physiological state on VBM results**. T1-weighted MPRAGE structural MRI scans were acquired in nicotine-dependent individuals after recently smoking (Smoke condition) or maintaining 5 h of monitored abstinence (No Smoke condition) and compared. Use of VBM to reveal gray matter indices showed significantly greater gray matter volume in the Smoke compared to the No Smoke condition in bilateral ventral striatum. Data are shown overlain on the Montreal Neurological Institute (MNI) template brain using neurological convention (left is left). X, Y, Z, coordinates shown are from the peak voxel; left, −22 6 −2 [143 voxels, *t*_(37)_ = 2.93, *p* = 0.006] and right, 28 8 −2, [423 voxels, *t*_(37)_ = 3.18, *p* = 0.003]. The color bar represents the *t-*values from the statistical maps (left, *T* = 3.09; right, *T* = 3.20).

## Funding

This work was supported by the National Institutes of Health, National Institute for Drug Abuse (Charles P. O'Brien 5P60DA005186; Teresa R. Franklin K01DA015426-02; Teresa R. Franklin R21DA025882; Teresa R. Franklin R01DA029845-01A1 and Teresa R. Franklin R01DA03039401A1) and a Teresa R. Franklin Investigator-initiated Research grant from Pfizer Pharma. The funding organizations had no role in study design, data collection and analysis, decision to publish, or preparation of the manuscript.

## Conflict of interest statement

The authors declare that the research was conducted in the absence of any commercial or financial relationships that could be construed as a potential conflict of interest.

## Supplementary material

Figure S1 **Illustration of the overlap between MP-RAGE and blood flow results**. Representative coronal [left above and below (enlarged)] and sagittal (right) images showing the regions of increased blood flow (blue) and the structural alterations (red) and the overlap between them (purple) in the sated vs. withdrawal conditions. *P*-value is set at 0.05 for display. Blood flow and structural data were analyzed in SPM8 and overlain on the MNI brain.
