# Supplementary figures and images for: Limitations of the use of the MP-RAGE to identify neural changes in the brain: recent cigarette smoking alters gray matter indices in the striatum
Source: Front Hum Neurosci. 2015 Jan 28;8:1052. doi: 10.3389/fnhum.2014.01052 (PMC4309115; doi:10.3389/fnhum.2014.01052)

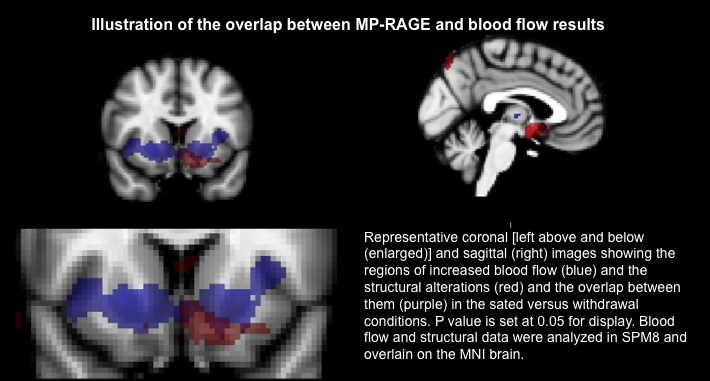

Supplement: Figure S1 — Illustration of the overlap between MP-RAGE and blood flow results. Representative coronal [left above and below (enlarged)] and sagittal (right) images showing the regions of increased blood flow (blue) and the structural alterations (red) and the overlap between them (purple) in the sated vs. withdrawal conditions. P-value is set at 0.05 for display. Blood flow and structural data were analyzed in SPM8 and overlain on the MNI brain. [file Image1.TIF]
